# Rapid pair-wise synteny analysis of large bacterial genomes using web-based GeneOrder4.0

**DOI:** 10.1186/1756-0500-3-41

**Published:** 2010-02-23

**Authors:** Padmanabhan Mahadevan, Donald Seto

**Affiliations:** 1 Department of Bioinformatics and Computational Biology, 10900 University Blvd., MSN 5B3. George Mason University, Manassas, VA 20110, USA; 2 Current address: Department of Biological Sciences, Vanderbilt University, Nashville, TN 37235, USA

## Abstract

**Background:**

The growing whole genome sequence databases necessitate the development of user-friendly software tools to mine these data. Web-based tools are particularly useful to wet-bench biologists as they enable platform-independent analysis of sequence data, without having to perform complex programming tasks and software compiling.

**Findings:**

GeneOrder4.0 is a web-based "on-the-fly" synteny and gene order analysis tool for comparative bacterial genomics (*ca*. 8 Mb). It enables the visualization of synteny by plotting protein similarity scores between two genomes and it also provides visual annotation of "hypothetical" proteins from older archived genomes based on more recent annotations.

**Conclusions:**

The web-based software tool GeneOrder4.0 is a user-friendly application that has been updated to allow the rapid analysis of synteny and gene order in large bacterial genomes. It is developed with the wet-bench researcher in mind.

## Findings

With the prospect now of very high-throughput and cost-effective DNA sequencing technology, and with its widespread applications routinely to many areas of biological interest, the number of available whole genome sequences grows at an astronomical rate. The Genomes Online Database (GOLD) [1] lists 957 complete and published bacterial genomes to date, with 3570 classified as "ongoing" (as of Dec. 2009). As a result of this tsunami of data, more genome analysis tools are needed in order to mine these data effectively, particularly for the bench scientist who may not be computer-savvy but is interested in using the genome data. Software tools have been developed for the analysis of gene order and synteny, which are important because they can be used to understand prokaryotic relationships and the evolution of their genomes [2], as well as to aid in gene function annotation [3], and to parse functional coupling prediction between genes in gene clusters [4]. In addition, new metrics are necessary in comparative genomics and systems biology to describe newly available sequenced genomes [5]; gene order and synteny may be used as one of these metrics to describe genomes.

Unfortunately, some of these useful and needed software tools have become software "orphans" and are no longer available or supported, for one reason or another [6]. In addition, some tools are not easily available to a wet-bench investigator as the tools must be downloaded, installed and compiled for use, *i.e*., problematic for non-computationally savvy users. In contrast, GeneOrder4.0 is a user-friendly, web-based tool that has been updated for the analysis of gene order and synteny between two large bacterial genomes. Previous versions of GeneOrder [7,8] were limited to viral and smaller bacterial genomes analyses (up to ~2 Mb), and have been useful since its inception; that is, as noted there are many bacterial species of interest that contain smaller genomes. The GeneOrder algorithm also enables the finding of "core" sets of genes are being used in a comprehensive re-survey of the relationships and taxonomy of the bacteriophages [9,10]. In response to requests, GeneOrder4.0 now performs rapid "on-the-fly" analysis of large bacterial genomes, up to at least 8 Mb size, with most analyses being completed between 5-10 minutes. This is achieved by implementing the "BLAST-like alignment tool" (BLAT) which performs more rapid "all-against-all" protein comparisons [11]. The result is a plot of protein similarity scores between two genomes highlighting gene order and synteny information, and proteins that have high similarity scores, based on user preference and input. Analyzing the proteins in common between two genomes gives insight into the "pan-genome" of sets of bacterial strains, which consists of these shared proteins and contrasts the unique proteins [12]. This will be useful, for example, in developing models for metabolic and expression regulatory pathways and for developing systems for bioremediation using a synthetic biology approach, one can re-annotate rapidly the genomes sequenced earlier using the more recently annotated ones. Thus, genomes such as the metal reducing bacteria, *Shewanella*, can be re-engineered and modelled based on newer genomes: *S. oneidensis *MR-1 (4657 genes), *Shewanella *sp. MR-4 (4099 genes), *Shewanella *sp.W3-18-1 (4238 genes) and *S. denitrificans *OS217 (3914 genes). Additionally, these four may be analyzed to map their metabolic pathways, allowing "gaps" to be either re-annotated or alternatives be examined.

### Implementation

GeneOrder4.0 is implemented using Java servlets and Tomcat. The tool requires entering two genomes as GenBank genome accession numbers. Files are retrieved from GenBank and the protein sequences are extracted. These sequences then serve as the input to BLAT algorithm. The speed of BLAT is due to the indexing of the database, whereas in the original BLAST, the query is indexed. Using BLAT allows the larger genomes, beyond 1-2 Mb, to be analyzed. Each protein pair is plotted on a graph according to user-specified BLAT threshold scores (default scores are "highest" (200+), "high" (100-200), and "low" (75-100)). These threshold scores are as follows: If the score is 200+, the proteins are very likely to be homologous; if the score is 100-200, they may not be homologous but are highly similar; and if the score is 75-100, they have limited similarity. In all cases, these results point to additional analyses, including wet-bench work, to verify homologies and similarities. Pairs of related proteins, as symbols on the graph, are "*hot-linked*", resulting in the opening of two browser windows comprising specific GenBank protein entries. The user can then examine the protein sequences in detail and determine directions for additional analyses.

**Figure 1:**
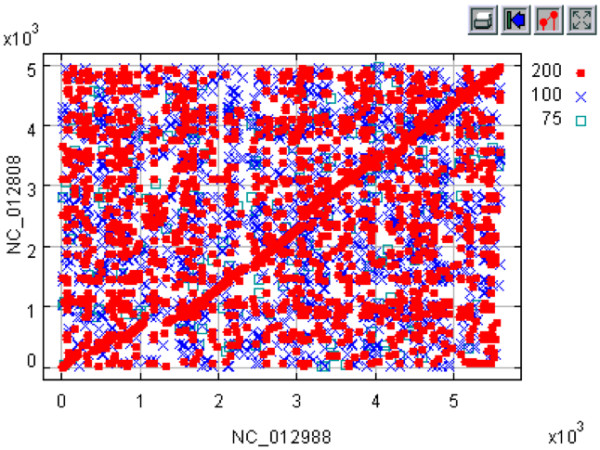
**Gene order plot between *M. extorquens *DM4 (**NC_012988) **and *M. extorquens *AM1 (**NC_012808). Default threshold settings are indicated in legend at the upper right, along with a tool bar for data visualization. Each point is linked to a pair of individual GenBank records.

Also in response to request, a plot applet now provides a higher degree of interactivity with the user. The first button in the group of four at the top right of the plot enables the user to print the plot (Figure 1). By clicking on the third button, the user can modify many aspects of the plot format such as the title and axes. Clicking and dragging on the plot allows the user to 'zoom in' to certain regions. The plot can be reset to its original appearance by using the second button, which resets the X and Y ranges to their original values. Finally, the last button enables rescaling of the plot to fit the data set size. These new features give the user more control over the presentation of the data, for viewing, analysis and publication. For example, if there are thousands of points on the graph and only small regions of synteny or inversion, the user can zoom in on those areas of interest and examine the protein pairs in greater detail. After examining these specific areas, the user can then restore the graph to the default "normal" view. In addition, the labels on the axes of the plot can be changed by the user for publication purposes. The user may wish to have the additional information such as the species name on the axes, as well as the GenBank accession number. Titles of the plot can also be changed to suit the user's needs (for example, "GeneOrder plot of species 1 vs. species 2"); this can easily be done by clicking the appropriate button on the plot applet. The color of the points on the plot can also be changed to black and white; this is an important feature because the charge of color pages in some journals can be high and therefore, the user can choose to remove color from the plot.

In addition to the graphical representation of gene order and synteny, a table of protein similarity scores is generated and can be viewed by clicking the link on the bottom of the plot. This table has four columns comprising the two genomes, the score and whether either of the proteins are "hypothetical" proteins. These are noted with "*" in the last column with the heading "Hypo?". This is a useful function for annotating hypothetical proteins if the corresponding protein has a function that has already been annotated, especially in more recently sequenced and annotated genomes. Again, this is more convenient for the lab-bench researcher who is not inclined to use more detailed software.

### Analysis of bacterial genomes using GeneOrder4.0

To illustrate a GeneOrder4.0 application in the analysis of a pair of *ca*. >4 Mb bacterial genomes, *Methylobacterium extorquens *DM4 (NC_012988) and *Methylobacterium extorquens *AM1 (NC_012808) were analyzed for gene order and synteny (5.9 Mb and 5.5 Mb, respectively). A two-dimensional plot (Figure 1) displays the results: red diagonal lines of similar protein pairs, indicated by red dots, indicate genome regions of synteny and high BLAST scores. Lower scores are indicated by crosses and open squares, all which may be default minimum values or user entered. The time it takes to generate this plot is approximately 4 minutes, a vast improvement over the earlier versions. As noted, an additional application of GeneOrder4.0 is for annotating hypothetical proteins. Many older genomes that were annotated in the past have coding sequences marked as hypothetical; these "holes" have been filled in for more recently sequenced and annotated genomes. However, in most cases, the GenBank file is not amended so the researcher retrieving the annotation often may have an annotation with many "hypotheticals", despite the fact that similar and more recent genomes, e.g., multiple and additional strains of the same organism, may have these particular "hypotheticals" annotated correctly. An experienced computational-savvy researcher may have other means to determine the updated annotations, but the 'casual' and often wet-bench researcher may not have the same experience. GeneOrder4.0 allows the alignment of these "hypotheticals" to the correct and presumable homologs or closest scoring equivalent, using specified BLAST/BLAT scores. As an example of this function, in *M. extorquens *DM4, the corresponding protein of a "hypothetical" based on BLAT score in *M. extorquens *AM1 is annotated as a putative protein-L-isoaspartate O-methyltransferase (GI:240141547). Additional and closer inspection of these two coding sequences show that these two proteins have the same length of 216 amino acids and share 97.6% identity, strongly suggesting that they have a conserved function. The GenBank entry for the hypothetical protein in *M. extorquens *DM4 (GI:254564064) does not mention that it may be a methyltransferase; hence, wet-bench verification is suggested from this putative annotation from GeneOrder4.0.

**Figure 2:**
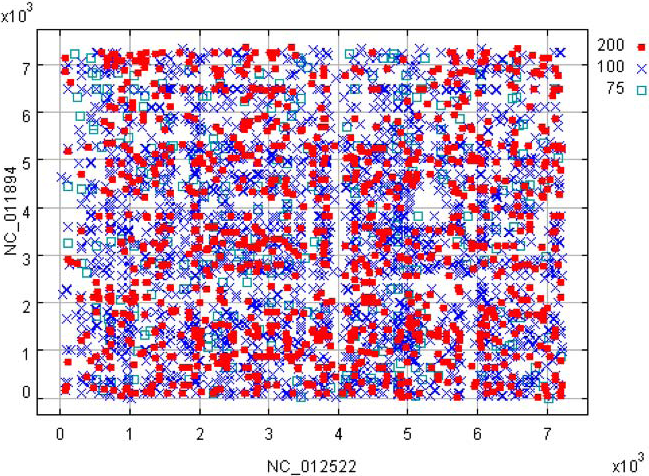
**Gene order plot between Rhodococcus opacus (**NC_012522) **and the Methylobacterium nodulans (**NC_011894). Default threshold settings are indicated in legend at the upper right, along with a tool bar for data visualization. Each point is linked to a pair of individual GenBank records.

**Figure 3:**
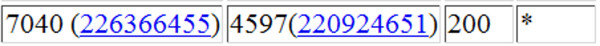
**Example of annotation of a hypothetical protein using GeneOrder4.0**. A row displaying computed paired similar proteins from the tabular output of GeneOrder4.0. The first column shows a protein belonging to *Rhodococcus opacus *and the second column shows a protein belonging to *Methylobacterium nodulans*. This match is a function of the default or user-entered threshold discrimination score, reflected in the third column, which shows the BLAT score. The fourth column indicates, by the presence of a "*", whether either of the proteins are annotated as hypothetical. Numbers in front of the links are the protein numbers, designated in numerical order from its Genbank entry, in the genome. By clicking the (underlined) links, GenBank files are displayed showing that the *Rhodococcus opacus *protein in the first column is annotated as hypothetical, while the *Methylobacterium nodulans *protein in the second column is annotated as a beta-lactamase domain protein.

Much larger bacterial genomes such as the 7.9 Mb *Rhodococcus opacus *(NC_012522) and the 7.7 Mb *Methylobacterium nodulans *(NC_011894) can be analyzed also using GeneOrder4.0, albeit with the analysis taking approximately 6 minutes (Figure 2). This is without the use of pre-computed and stored similarity scores and is performed "on-the-fly", allowing the analysis of novel genomes. One reason for this is that some researchers are wary of having their proprietary data stored and archived. There are surprisingly few proteins annotated as "hypothetical" in these two genomes, perhaps due to the improvement of gene annotation techniques as they are relatively recently sequenced genomes. Nevertheless, one example may be seen in the *R. opacus *genome, where a coding sequence is annotated as "hypothetical" (GI:226366455) (Figure 3). Its putative counterpart in *M. nodulans *is annotated as a beta-lactamase domain protein (GI:220924651), both having similar lengths (data not shown) and a percent identity of 42.8%. Upon further computational analysis using ProDom [13], it was found that the hypothetical protein also has a beta-lactamase domain, suggesting that these two proteins may have similar functions. Of course, final confirmation lies in performing wet-bench experiments, but this computational analysis using GeneOrder4.0 provides a valuable clue and a starting point as to the putative function of the hypothetical protein, and suggests additional computational analyses for unraveling the identity, again with the wet-bench researcher in mind.

User-friendly and web-accessible tools allow wider access and application of the information-rich burgeoning data in genome databases. For many wet-bench biologists, again, this is very convenient because no downloading and installing of software are required; no compiling or tinkering with code is necessary. In addition to the enhancements for data visualization and graphing, the major advantage of GeneOrder4.0 is that it performs rapid analysis of two large bacterial genomes, while retaining its original validated "easy to use" functionality for the pair-wise gene order and synteny analysis of smaller genomes (mitochondrial, chloroplast, viral and bacterial).

## Availability and requirements

• **Project name**: GeneOrder4.0

• **Project home page: **http://binf.gmu.edu:8080/GeneOrder4.0

• **Operating system(s): **Platform independent

• **Programming language: **Java

• **Any restrictions to use by non-academics: **Contact authors

## Competing interests

The authors declare that they have no competing interests.

## Authors' contributions

PM implemented and validated the software. PM and DS conceived and wrote the paper.
